# 靶动脉栓塞化疗联合氩氦刀等微创技术治疗原发性非小细胞肺癌139例分析

**DOI:** 10.3779/j.issn.1009-3419.2010.01.11

**Published:** 2010-01-20

**Authors:** 凌飞 罗, 洪武 王, 洪明 马, 珩 邹, 冬妹 李, 云芝 周

**Affiliations:** 100028 北京，北京煤炭总医院肿瘤微创治疗中心 Minimal Invasive Tumor Terapy Center, Meitan General Hospital, Beijing 100028, China

**Keywords:** 栓塞化疗, 氩氦刀, 原发性, 肺肿瘤, Chemoembolization, Cryosurgery, Primary, Lung neoplasms

## Abstract

**背景与目的:**

动脉内栓塞化疗、氩氦刀靶向冷冻及放化疗粒子植入是目前治疗肺癌的主要微创技术，本文通过对患者治疗后生存质量、临床有效率和生存期进行总结，并对每项技术自身优势和应用局限性进行分析。本研究旨在探讨多种微创治疗技术联合应用治疗晚期非小细胞肺癌的临床疗效。

**方法:**

回顾性分析2006年7月-2009年7月经病理证实并完成随访的139例患者，综合评价均已失去外科手术切除机会。其中原发病灶102个，纵隔、肺内及胸壁转移病灶37个，依据病灶血供情况及病灶大小、位置等选择不同微创治疗技术组合，其中靶动脉超选择栓塞化疗、氩氦刀靶向冷冻及放化疗粒子植入相结合治疗富血肿瘤71个，单纯氩氦刀靶向冷冻治疗乏血肿瘤48个，氩氦刀靶向冷冻结合放、化疗粒子植入治疗乏血肿瘤20个。对患者治疗前后KPS评分、影像资料及随访结果进行对比分析。

**结果:**

治疗后患者KPS评分平均提高20.01，随访3年，CR 44例，PR 87例，NC 3例，PD 5例，有效率94.2%。1年生存99例（71.2%），2年生存43例（30.2%），4例存活3年以上，中位生存19个月，平均生存（16±1.5）个月。无脊髓损伤、血管及心包穿刺损伤等严重并发症。

**结论:**

微创技术操作成功率高、创伤小、并发症轻、疗效肯定。就原发性非小细胞肺癌而言，根据患者具体情况，有针对性地采用不同微创技术相结合实施治疗，相互补充、协同作用，将进一步提高患者中、远期临床疗效。

## Abstract (en)

**Background and objective:**

TACE, Ar-He target cryosurgery and radioactive seeds implantation are the mainly micro-invasive methods in the treatment of lung cancer. This article summarizes the survival quality after treatment, the clinical efficiency and survival period, and analyzes the advantages and shortcomings of each methods so as to evaluate the clinical effect of non-small cell lung cancer with multiple minimally invasive treatment.

**Methods:**

All the 139 cases were nonsmall cell lung cancer patients confirmed by pathology and with follow up from July 2006 to July 2009 retrospectively, and all of them lost operative chance by comprehensive evaluation. Different combination of multiple minimally invasive treatments were selected according to the blood supply, size and location of the lesion. Among the 139 cases, 102 cases of primary and 37 cases of metastasis to mediastinum, lung and chest wall, 71 cases of abundant blood supply used the combination of superselective target artery chemotherapy, Ar-He target cryoablation and radiochemotherapy with seeds implantation; 48 cases of poor blood supply use single Ar-He target cryoablation; 20 cases of poor blood supply use the combination of Ar-He target cryoablation and radiochemotheraoy with seeds implantation. And then the pre- and post-treatment KPS score, imaging data and the result of follow up were analyzed.

**Results:**

The KPS score increased 20.01 meanly after the treatment. Follow up 3 years, 44 cases of CR, 87 cases of PR, 3 cases of NC and 5 cases of PD, and the efficiency was 94.2%. Ninety-nine cases of 1 year survival (71.2%), 43 cases of 2 years survival (30.2%), 4 cases with over 3 years survival and the median survival was 19 months. Average survival was (16±1.5)months. There was no severe complications, such as spinal cord injury, vessel and pericardial aspiration.

**Conclusion:**

Minimally invasive technique is a highly successful, micro-invasive and effective method with mild complications. To non-small cell lung cancer, we can improve the middle and long term clinical effect by using the different combination of multiple minimally invasive treatments according to the patient's condition.

近些年随着医学的进步，各种微创治疗技术不断出现，主要包括靶动脉栓塞化疗、氩氦刀靶向冷冻、放化疗粒子植入和射频消融等。每种治疗技术既有自身优势，又有应用局限性。因此，针对不同病例选择不同的微创治疗技术联合应用将是提高晚期恶性肿瘤患者远期临床疗效的关键。

## 1 资料与方法

### 1.1 主要资料

2006年7月-2009年7月共治疗158例患者，失访19例。完成随访的139例中男性78例，女性61例，平均年龄64.8岁。Ⅲb期41例，Ⅳ期98例。包括鳞癌69例，腺癌59例，腺鳞癌11例。治疗前疼痛指数5以上者39例，平均KPS评分60±11.2。

### 1.2 适应证及操作方法

#### 1.2.1 靶动脉栓塞化疗(TACE)

Seldinger技术穿刺股动脉，5fCobra(C3)或西盟导管行靶动脉超选择插管，成功后使用微导管做进一步超选择插管。肿瘤供血动脉主要包括支气管动脉、肋间动脉、胸廓内动脉及甲状颈干分支等体动脉。化疗方案VP-16+DDP、GEM+DDP。32(71)例使用液态碘化油(3 mL-5 mL)+ADM(20 mg-30 mg)混合成乳剂栓塞肿瘤血管床，全部患者均使用明胶海绵微粒栓塞肿瘤供血动脉主干。

#### 1.2.2 氩氦刀靶向冷冻(Ar-He cryosurgery)

冷刀头包括2 mm、3 mm、5 mm和8 mm，相应形成的治疗冰球大小依次为2 cm-3 cm、5 cm-6 cm、7 cm-8 cm和9 cm-10 cm。依据影像资料明确靶区并设计三维治疗计划，要求治疗冰球大于病灶边缘1 cm，单次治疗冷冻范围达病灶80%以上为显效。CT引导下无菌操作，局麻下穿刺到位后经外鞘管置入冷刀，多刀同时启动冻融治疗循环，每个循环冷冻15 min+升温5 min，一般单次治疗需2个冻融循环。如肿瘤较大时单次治疗不足以达显效，可采用退刀冷冻或分次冷冻。治疗全过程均需监测患者心率、血压、血氧饱和度等生命体征。

#### 1.2.3 放/化疗粒子植入(radioactive seeds and releasecontrolled chemical drugs implantation)

属同步放、化疗。依据影像资料明确靶区并设计三维治疗计划，CT引导下无菌操作，局麻下专用粒子植入针穿刺到位后依计划植入放、化疗粒子。目前国内使用的放疗粒子为^125^Ⅰ，每枚0.8 mCi，辐射范围1 cm^2^，每60天剂量衰减一半，有效作用时间90 d，每次治疗植入^125^Ⅰ粒子总量不超过40枚。缓释化疗药粒子主要有顺铂(中人顺安)和5-氟尿嘧啶(中人氟安)，肺癌主要使用中人顺安(20 mg/3支、瓶)。穿刺针间隔1 cm，放、化疗粒子交叉植入，间隔0.5 cm。

#### 1.2.4 适应证

全部患者治疗前行CT增强扫描，依据病灶血供情况、位置、大小以及穿刺风险评估选用不同的微创治疗组合。靶动脉栓塞化疗+氩氦刀+粒子植入适应证：病灶富血且大小 > 5 cm；病灶富血且大小 < 5 cm但位置临近大血管、主气管、心包和体表。靶动脉栓塞化疗+氩氦刀适应证：病灶富血且大小≤5 cm的周围型肿瘤。靶动脉栓塞化疗+粒子植入适应证：病灶富血但位置隐匿，穿刺风险大，如纵隔内肿瘤。氩氦刀+粒子植入适应证：病灶乏血，大小 > 5 cm或病灶 < 5 cm但位置临近大血管、主气管、心包和体表。单纯氩氦刀适应证：病灶乏血，大小≤5 cm的周围型肿瘤。单纯粒子植入适应证：病灶乏血且位置隐匿，穿刺风险大，如纵隔内肿瘤。

本组治疗其中原发病灶102个，纵隔、肺内及胸壁转移病灶37个。富血肿瘤71个，靶动脉超选择栓塞化疗+氩氦刀靶向冷冻+放化疗粒子植入44个，靶动脉超选择栓塞化疗+氩氦刀靶向冷冻16个，靶动脉超选择栓塞化疗+放化疗粒子植入11个，乏血肿瘤68个，氩氦刀靶向冷冻+放化疗粒子植入20个，单纯氩氦刀靶向冷冻48个。首次治疗完成后需根据患者恢复情况确定后续治疗时间，一般间隔3天-10天。

## 2 结果

全部操作技术成功率100%。与操作有关的并发症包括2例栓塞化疗术中出现一过性脊髓缺血症状，表现为单侧足、趾麻木，10 min-20 min后自行缓解。气胸19例，肺内出血21例，均在3-7日内治愈，无脊髓损伤、大血管、心包损伤以及交通性气胸等严重并发症。

39例有明显疼痛症状患者，主要因为肿瘤累及胸膜或有骨质破坏所致，治疗后21例疼痛即刻缓解，18例1天-3天后明显缓解。全部患者治疗后KPS平均提高20.01。

随访3年，CR(complete relief, CR)44例，全部为单纯氩氦刀和氩氦刀联合粒子植入治疗的患者，其中肺内原发肿瘤39个，肺内转移瘤5个，均为乏血病灶。其他患者PR(partial relief, PR)87例，NC(normal control, NC)3例，PD(progresive disease, PD)5例，有效率94.2%。就总生存期(overall survival, OS)而言，6个月生存131例(94.2%)，1年生存99例(71.25%)，2年生存43例(30.2%)，4例存活3年以上。中位生存19个月，平均生存(16±1.5)个月。Ⅲb期41例，OS均超过1年，35例OS超过2年。Ⅳ期98例，58例OS超过1年，8例OS超过2年。

典型病例1：72岁，腺癌，富血。TACE联合氩氦刀冷冻治疗，OS为26个月(图 1)；典型病例2：63岁，腺癌，乏血。单纯氩氦刀冷冻治疗，OS为18个月(图 2)；典型病例3：68岁，鳞癌，乏血。氩氦刀联合粒子植入治疗，OS为13个月(图 3)；典型病例4：64岁，鳞癌，富血。TACE联合粒子植入治疗，OS为14个月(图 4)。

**1:**
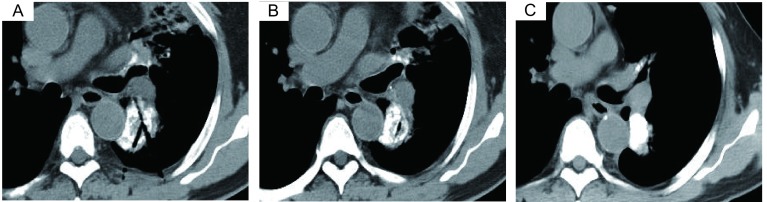
周围型富血肺癌，TACE联合氩氦刀治疗 Peripheral lung cancer with plenty blood supply, TACE combined Ar-He cryosurgery

**2:**
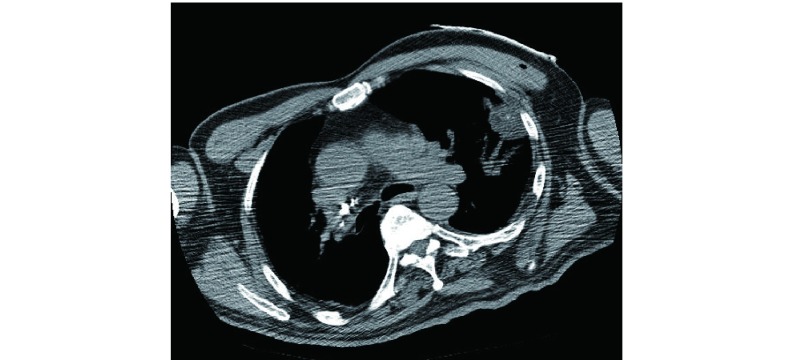
周围型乏血肺癌，肿瘤 < 5 cm，单纯氩氦刀靶向冷冻治疗 Peripheral lung cancer with lack blood supply, less than 5 cm, simple Ar-He target cryosurgery

**3:**
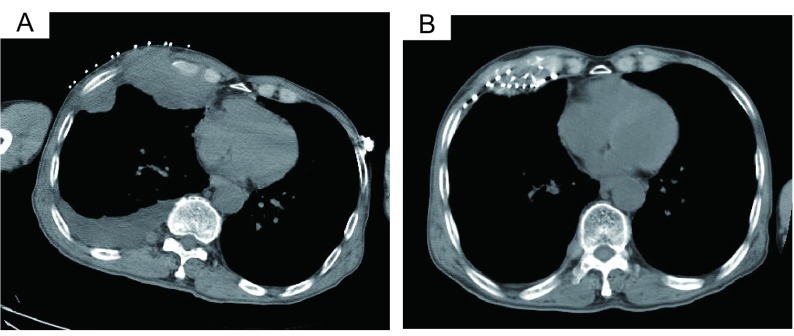
肺癌胸壁转移，氩氦刀联合放/化疗粒子植入治疗 Lung cancer with metastasis of chest wall, Ar-He cryosurgery combined with radioactive seeds implantation

**4:**
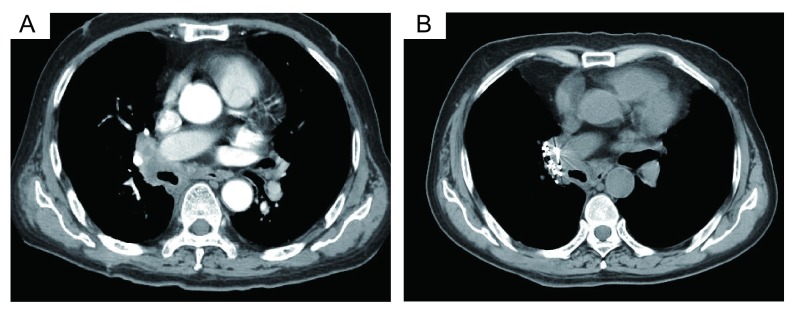
中央型富血肺癌，TACE联合放、化疗粒子植入治疗 Central lung cancer with plenty blood supply, TACE combined radioactive

## 3 讨论

原发性非小细胞肺癌在肺癌中所占比例约为70%-80%，对全身放、化疗敏感性差，加之确诊时多属晚期而失去外科手术切除机会。支气管动脉灌注化疗虽在一定程度上提高了临床疗效，但近期有效率也仅为50%。支气管动脉栓塞化疗近期疗效满意，但远期疗效明显受到限制。首先，非小细胞肺癌中以周围型为主的乏血肿瘤占相当比例，单纯栓塞化疗很难获得满意疗效。其次，每次的动脉内操作和栓塞都会不同程度的损伤肿瘤靶血管，导致靶血管显示率减低，超选择插管难度加大，事实上可以让医生实施有效治疗的次数非常有限。因此，支气管动脉栓塞化疗在很长一段时期仅仅是一种辅助性治疗手段。比如传统外科手术前的新辅助化疗可增加手术切除率。近些年，氩氦刀靶向冷冻、放化疗粒子植入以及射频消融等微创治疗技术的出现，使我们得以重新认识靶血管栓塞化疗的价值。我们体会，靶血管栓塞化疗与其它微创治疗技术结合，其最明显的价值在于：肿瘤血管有效栓塞阻断瘤体内血液循环，抑制了氩氦刀治疗过程中的热池效应，明显提高冷冻效果；减少穿刺并发症；靶血管栓塞化疗使巨大肿瘤在近期内缩小，提高单次冷冻治疗率；肿瘤缩小还使冷冻靶区容易控制，减少肿瘤周边正常结构损伤。截至目前，多数医生对碘化油的使用持谨慎态度，我们的实践显示碘化油+化疗药乳剂栓塞肿瘤血管床可明显增加栓塞化疗效果，前提是：确保微导管插管准确、到位，避开脊髓动脉；肿瘤内不存在动-静脉瘘；透视监视下经微导管缓慢推注。

氩氦刀靶向冷冻是以氩气为冷源、氦气为热源的冻融治疗技术，其核心是快速降温使细胞内产生冰晶，复温则使冰晶爆裂导致细胞变性坏死。在细胞被摧毁过程中，最低冷冻温度、细胞内冰晶形成速度、冷冻时间以及冻融循环次数是决定因素。王洪武教授研究显示，零下40 ℃-零下50 ℃时，细胞死亡率最高，可达40%-60%，而温度进一步降低，细胞死亡率变化不明显。当温度从零下100 ℃复温至零下20 ℃时，细胞内冰晶爆裂，细胞死亡率进一步增加，所以冻融治疗比单一冷冻和单一热疗更具有摧毁性，一般两个冻融循环可使靶区内细胞完全坏死。肿瘤三维治疗计划、靶区控制及肿瘤内血液循环的存在明显影响治疗效果，因为每把冷刀所形成的冰球，其内部降温效果并不一致，冰球边缘往往达不到所要求的低温，因此， < 2 cm病灶可采用单刀冷冻，而 > 2 cm的病灶都需要多刀组合，才能使最低有效温度完全覆盖靶灶，从而杀灭全部肿瘤细胞，避免肿瘤边缘复发。靶区控制可避免冷冻范围过大，以最大限度保护病灶周围正常结构，减轻或减少并发症发生。肿瘤内丰富的血液循环会明显增加热池效应，使冷冻不能在瞬间降低至有效温度，肿瘤细胞“保鲜”而不是坏死。就此而言，肿瘤靶血管栓塞则尤为关键。对于 < 2 cm的周围型乏血肿瘤，氩氦刀靶向冷冻可获得与外科手术同样的治疗效果。对于 < 5 cm的周围型乏血肿瘤，单纯氩氦刀靶向冷冻即可完全杀灭肿瘤细胞，1年生存率可达60%。对于 > 5 cm的肿瘤，则需要退刀冷冻或分次冷冻。对于临近体表、心包、大血管、主气管的肿瘤，单纯氩氦刀治疗则很难完全杀灭肿瘤细胞，需结合放、化疗粒子植入做后续治疗。

局部放疗加全身化疗的同步治疗已被广大学者认可，但严重的并发症及副反应明显限制其临床疗效，如皮肤损伤、放射性肺炎、气管、食管穿孔以及化疗药副反应等。此外，如果肿瘤位置深，周围正常结构丰富，再加上逐层衰减，则很难使靶灶获得有效治疗剂量。放、化疗粒子植入是一种后装示组织间插植技术，属近距离同步放、化疗。依照三维植入计划植入放、化疗粒子，放疗粒子可长时间作用于靶灶，化疗粒子缓慢、持续释放则可保证靶灶内长时间高浓度作用。放、化疗粒子同时植入既保证了有效的治疗作用，又对靶灶临近结构无损伤。我们体会粒子植入的穿刺风险远低于氩氦刀靶向冷冻，但单纯依靠粒子植入，治疗费用偏高。目前粒子植入主要适用于 < 3 cm的靶灶；靶灶位置隐匿，穿刺风险大，如纵隔内或皮下肿瘤；续贯氩氦刀和/或栓塞化疗，以控制残留病灶。

本组患者治疗后，临床症状缓解，生存质量提高、生存期延长。随访3年，CR 44例，PR 87例，NC 3例，PD 5例，有效率94.2%。6个月生存131例(94.2%)，1年生存99例(71.2%)，2年生存43例(30.2%)，4例存活3年以上。中位生存19个月，平均生存(16±1.5)个月。

总之，微创技术操作成功率高、创伤小、并发症轻、疗效肯定，每项技术既有其优势，也有不足和应用局限性。就原发性非小细胞肺癌而言，根据每个患者具体情况，包括全身状况、肿瘤血供情况以及肿瘤大小、位置等，选择合理的微创治疗组合，既符合综合、续贯治疗原则，也使得各种治疗手段能够相互补充、协同作用，必将进一步提高患者中、远期临床疗效。在多种微创治疗技术联合应用的前提下，是否可放弃动脉内大剂量的化疗药灌注值得进一步研究。
